# Announcement: Graduate Research Seminar in Bioinorganic Chemistry

**DOI:** 10.1155/MBD.1997.302

**Published:** 1997


					GRADUATE RESEARCH SEMINAR
IN BIOINORGANIC CHEMISTRY
23-26 January, 1998,
Harbortown Marina Resort, Ventura, California, USA
This is a Gordon Research Conference designed to bring together graduate students, postdoctoral
researchers, and young scientists conducting research in bioinorganic chemistry. This is a unique
opportunity for young scientists to see current and innovative work and to present their work to the
scientific community in a relaxed environment comprised of peers and future collaborators. Sessions
will be lead by established researchers in the field, promoting cross talk and in-depth discussion of
papers. All researchers in the broadly defined area of bioinorganic chemistry are encouraged to
apply. Oral and poster presentations by graduate students and postdoctoral researchers in the field
of bioinorganic chemistry will be organised. A joint session with Metals in Biology GRC (Thursday
evening) featuring a seminar by Prof. Joan Valentine (UCLA): "Searching for the link between Cu-Zn
superoxide dismutase and ALS" and a keynote seminar at GRSBI: Prof. Brian Hoffman
(Northwestern): Some specific areas which are likely to be covered are:
Metalloproteins/ Metalloenzymes:
mechanistic studies, structure/function relationships, model systems of metalloprotein active sites,
biological electron transfer, metalloprotein folding
Metals in gene regulation or drug therapy
Metal-nucleic acid interactions
Abstracts are requested. The exact content of the conference will be organized around the papers
submitted. Papers not selected for oral presentation will be presented during one of three poster
sessions. One of these poster sessions will be a joint session with the Metals in Biology conference
on the first evening of the GRSBI.
FOR MORE INFORMATION:
1) Go to The GRC home page: http://www.grc.uri.edu
or email the GRC at grc@grcmail.grc.uri.edu
2) Contact one of the co-chairs of the conference:
Please email to receive a second announcement. Include your fax, mail and email address.
Conference Co-Chairs:
Carol M. Gorst, Department of Chemistry, University of Michigan, Ann Arbor, MI 48109, USA
gorstcm@ umich.edu
Shana O. Kelley, California Institute of Technology, Division of Chemistry and Chemical
Engineering, Pasadena, CA 91125, USA
kelleysh @ cco.caltech.edu
General Information:
Registration Fees:
$380 single room
$320 double room
$245 for attendees lodging offsite
This fee includes hotel room, dining for the duration of the conference, and all conference fees
Partial travel reimbursements will be awarded to as many of the conference attendees as possible.
Additional information will be supplied in a second announcement.
302

				

